# Geochemical, Mineralogical, and Geomorphological Characterization of Ash Materials as a Tracer for the Origin of Shifting Sands near Oldupai Gorge, Ngorongoro, Tanzania

**DOI:** 10.1155/2022/2593944

**Published:** 2022-10-28

**Authors:** Mohamed Zengo Makongoro, Maheswara Rao Vegi, Said Ali Hamad Vuai, Michael Mwita Msabi

**Affiliations:** ^1^Laboratory Directorate, Geological Survey of Tanzania, P.O. Box 903, Dodoma, Tanzania; ^2^Department of Chemistry, College of Natural and Mathematical Sciences, The University of Dodoma, P.O. Box 259, Dodoma, Tanzania; ^3^Department of Geology, College of Earth Sciences and Engineering, The University of Dodoma, P.O. Box 259, Dodoma, Tanzania

## Abstract

Shifting sand (SS) is a single dune-shaped mass of black ash material moving across western Ngorongoro in northern Tanzania. The moving sand has become an important tourist destination for several decades. Despite being part of the important geosites at the Ngorongoro Conservation Area, the nature, origin, and behaviors demonstrated by SS remain poorly understood. This work contributes toward understanding the nature and identification of the possible origin of the SS through the correlation of geochemical, mineralogical, and geomorphological data of ash material from four selected locations in the study area. To achieve this goal, elemental, mineralogical, and morphological characterization of ash samples was performed by energy-dispersive X-ray fluorescence, polarized petrographic microscopy, automated sieve shaker, and binocular microscopy techniques, respectively. Correlation studies were based on magnesian-ferriferous associations, similarities in mineralogy, particle size, shape, and distribution patterns of ash materials, and weather data. There are close similarities in the chemical compositions among ash samples of SS, Ootun area, and Oldoinyo Lengai. Augite and magnetite minerals appear only in samples of SS, Ootun area, and Oldoinyo Lengai, while hornblende appears only in the samples from the Ngorongoro crater. Oldoinyo Lengai rock petrography revealed significant amounts of augite minerals. Blocky and elongated-shaped ash particles dominate the samples from SS, Ootun area, and Oldoinyo Lengai. The particle size of ash materials decreases westwards across the study site. The distribution patterns of ash material align with the west-south-west wind direction. Based on these findings, the study concludes that SS and Ootun ash could be tephra depositions resulting from past volcanic eruptions of Oldoinyo Lengai.

## 1. Introduction

Sand dunes are well-known in desert areas [1]. The structures of dunes are made up of sand masses of different morphologies as a result of prevailing winds. There are two types of dunes, namely, linear dunes, and barchans, which are formed by fluid dynamics at the particle level above sand surfaces that explain the relationship of sand particles with the wind [2]. The size of sand particles is essential in determining morphological fate. Particles with a diameter of less than 0.050 mm are deemed cohesive, thereby resisting erosion by wind. On the other hand, particles with diameters between 0.062 and 2.0 mm are easily carried by the wind due to their noncohesive nature. This property explains why sand dunes are composed of sand particles of 0.125 to 0.250 mm [2].

It has generally been known that dunes are the geomorphologic signatures of wind-surface interactions [3]. Their shape, size, direction, and orientation result from complex wind dynamics involving several factors that should not be viewed simplistically [1]. These factors may include the accretion rate of particles, erosion loss of surface particles, turbulence, deposition, and accreted dust [4].

The genesis of a dune landform from an unstable surface has been explained by steady-state theory [2]. Steady-state theory (waveform theory) defines the stable nature of the dune landform while advancing. It explains the equilibrium that exists between the wind and the resistance of the sand particles on the surface of the dune landform to cancel wind energy, thereby adjusting the dune shape to balance the effect. The balance is achieved by deflection of the incident wind from the tail side that spreads sand particles parallel to the crest, thereby shaping the horns on the leeward side. At this stage, the dune is said to be in a steady state [1, 2].

**Figure 1:**
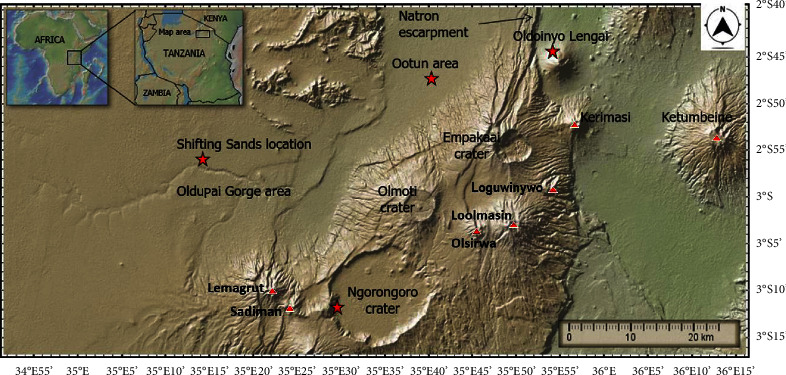
A map of part of the northern Tanzanian sector of the Gregory Rift was modified by the GeoMapApp global multiresolution topography [14], to show the study sites. The red star points are the four sampling locations.

Volcaniclastic dunes, on the other hand, are extremely rare on the Earth [5]. Their occurrences have primarily been observed in the Ka ‘u Desert in Hawaii, United States of America, which is thought to have been derived from Kilauea volcanic eruptions [6]. Volcaniclastic dunes have also been observed in parts of New Zealand and Iceland [5]. Northern Tanzania is endowed with several unique features of geological significance [7–13], including substantial amounts of volcaniclastic ash material scattered across the rift valley enclave at the Ngorongoro Conservation Area (NCA) spreading from Oldoinyo Lengai (OLA) to Oldupai Gorge (Figure 1) at varying amounts, shapes, and behavior.

**Figure 2:**
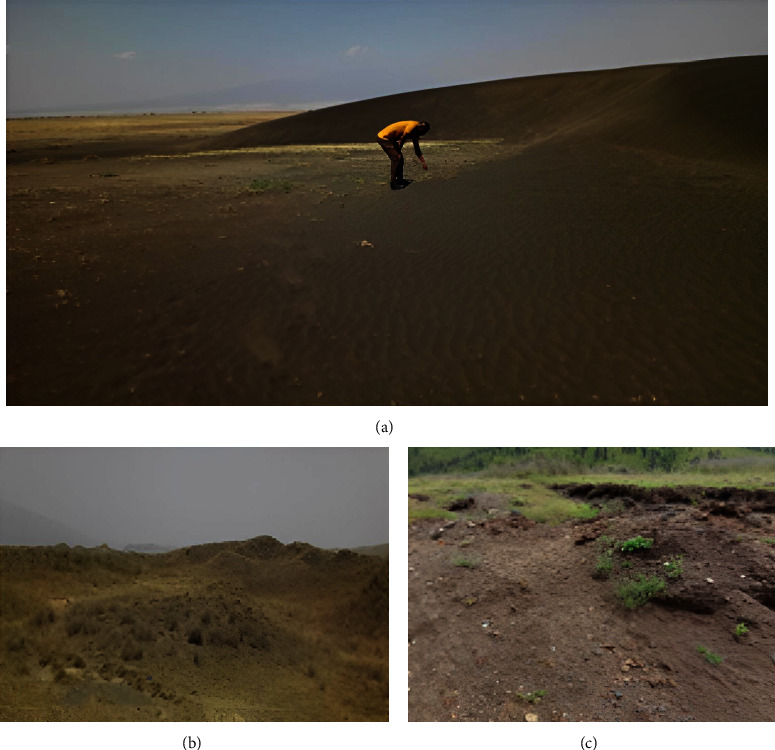
(a) Shifting sands (SSs) near Oldupai Gorge area, (b) Ootun sands (OTSs), and (c) the Ngorongoro Conservation Area (NCA) sampling site (source: field survey photographs by the research team with permission, September 2020).

**Figure 3:**
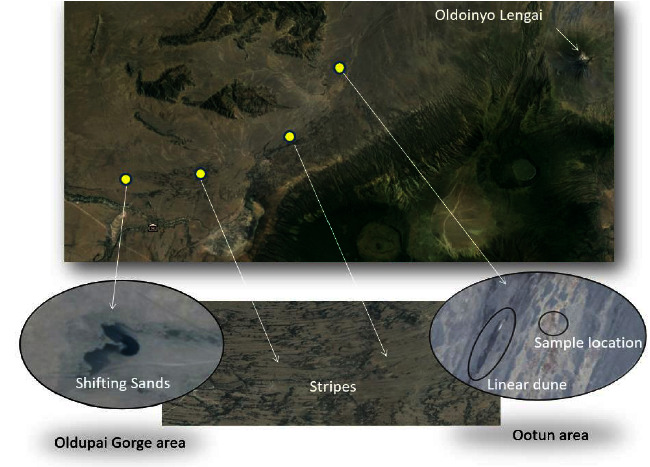
The image from the landscape across the study area shows around 84 km wide, with the shifting sands (SSs) in the west, Ootun sands (OTSs) at the center, and Oldoinyo Lengai in the east (source: Google Earth satellite).

There are two types of ash bedforms on the study site. These are migrating dunes known as shifting sands (SSs) and vegetation-covered stationary bedforms known as Ootun sands (OTSs), located about 35 kilometers west of OLA (Figures 2(a) and 2(b)). The area is characterized by vegetative piles made up of ash material that has been deposited in the past from the Gregory Rift volcanoes. The Google Earth landscape across the study area is shown in Figure 3. The coordinates of SS in Google Earth Pro are 2°56′32″S 35°18′42″E, and for OTS, they are 2°46′44″S 35°36′16″E.

Black ash material, which is a barchan dune famed as shifting sands (Figure 2(a)), is located near Oldupai Gorge, about 80 km west-south-west of OLA [15] (Figure 1). The SS is a single mass of ash bedforms moving slowly in a westward direction across the western Ngorongoro plains at a rate of about 16 to 20 meters per year. Normally, its size is about 3.5 meters high, 28 to 36 meters wide, and 10 meters from the windward to the leeward side. It is composed of unique fine black ash particles of unknown parentage of varying sizes and shapes. For many years, SS has been thought to be a bedform whose structure is likely to be modified by the wind. It has been a tourist destination for many decades as a unique structure, featuring many spiritual narratives on its behavior by the local Maasai communities around it.

Volcanogenic bedforms result from tephra depositions [16, 17]. Tephra is any pyroclastic material ejected during an explosive volcanic eruption regardless of grain size, composition, grain shape, or origin [18–21]. Their sizes, shapes, mobility, and distance from the source volcanoes are primarily determined by climatic conditions [18]. They are embedded with rich geological information associated with volcanism and can be interpreted to ascertain events in the distant past [21]. The ash material of SS is a subset of tephra, by definition consisting of particles with a diameter <2 mm [14]. The review in [22] points out the accuracy of using tephra to link and synchronise geochemical data and transfer them from one site to another. With the aid of credible and reliable chronology techniques, tephrochronological studies involve data correlation of tephra from different locations and ultimately come up with scientific estimations of the nature and origin of materials at a high degree of certainty [23, 24].

Tephra geochemistry remains among the strongest stratigraphic markers of the Holocene and Pleistocene [23–26]. The chemistry of volcanic ash has been applied as a tool for differentiating between volcanic bedforms and has been used as a historical marker for elucidating conditions and events that took place during and after volcanic eruptions [24–28]. This approach has essentially been important in addressing the source volcanoes of respective volcanic ash through tephra correlation studies and the geology of surrounding volcanoes. Nowadays, the interpretation of geochemistry, stratigraphy, and chronology data has been central to ascertaining desired information on archaeology, petrogenesis, chronostratigraphy, and explosive volcanisms with their time-space relationships [23]. These studies are essential in uncovering geological events as well as risk management and hazard predictions [25, 29].

Ash chemistry is related to magma chemistry and is influenced by processes following magma fragmentation, such as heterogeneous interactions in the eruption plume [30, 31]. Volcanic ash is typically dominated by the volcanic glass with lesser amounts of crystalline minerals and hence, in some instances, difficult to classify based on its mineralogical composition [32]. Ash and rock types around volcanoes can be correlated to reveal a source volcano of particular distal ash through the total alkali-silica (TAS) diagram, CaO versus K_2_O plot [23, 33], and various geochemical correlation tools as described in [34].

The attention to the movement of SS had only been paid from 1969 by recording its positions every year even though SS has existed for several decades. There is only one published work on the geochemistry of SS which is by Kafumu [35], who compared his results with the geochemistry of OLA rocks published by Bell and Simonetti [36]. In his study, Kafumu [35] found similarities between some elements in SS and rocks from OLA. However, detailed evidence from the ash comparison and a multidisciplinary approach to potential ash sources in the region are necessary to understand the nature and origin of the Shifting Sands. In this study, multidisciplinary techniques and critical analysis of the composition and origin of SS were adopted to investigate its nature and origin. Similar ash materials from other parts of the region were investigated for correlation purposes and processed by various geochemical tools, including those described in [32].

## 2. Material and Methods

### 2.1. Sampling

Black ash material from SS, OTS, OLA, and from the lower part of descending crater road at NCA (Figures 1 and 2(c)) was sampled. A total of 32 ash samples were taken as follows: eight samples from different parts of SS (horns, top, leeward, and windward sides of the dune) and a similar number of ash samples from OTS, OLA, and NCA. Great care was taken to ensure that the right representation of the material was taken. Each sample weighs about 1 kg. Each pooled sample was homogenized and reduced to about 250 g by the quartering method. The samples were taken for elemental, mineralogical, particle size, shape, and loss on ignition (LOI) analyses. Four samples of igneous rocks (ijolite) from OLA were also taken for petrographic studies. OLA is an active volcano and is thought to be the most likely source volcano of ash material. OTS contains stabilized ash dunes and is located about 35 km west of OLA. Samples from NCA were used as a control since they came from the Ngorongoro volcanic center, which, unlike OLA, ceased its activities more than 2 million years ago [37].  Table 1 shows a list of all samples.

### 2.2. Elemental Composition

Ash samples from the four sites were dried at 105°C to constant weight, well homogenized, and pulverized to 75 *µ*m particle size and analyzed as in [16]. The energy dispersive X-ray fluorescence (EDXRF) spectrometer (Model XL3t) was used to estimate the elemental composition of the samples. The EDXRF instrument is designed for carrying out analytical work in atmospheric conditions and helium gas environments. When analyzing light elements (with an atomic number less than 11), the instrument automatically opens helium gas to enhance detection. The rest elements are analyzed in the atmospheric conditions. The EDXRF measuring time (live time) was set at 100 s. Samples at 75 *µ*m were pressed into cups and kept in a sample compartment and read. The machine was equipped with Au and Ag anodes and a high-performance semiconductor detector. The samples were scanned at a maximum of 50 kV and 40 *µ*A while enhanced with external helium gas for light element detection. The Certified Reference Standards (DC73026) from the China National Analysis Centre for Iron and Steel (Beijing) were used to validate the data. The analyses were performed at the Geological Survey of Tanzania.

### 2.3. Loss on Ignition

Loss on ignition (LOI) for ash samples was determined by using the Nabertherm muffle furnace (Model L-241K2TN). A smaller particle size (75 *µ*m) enhances each part of the material to be exposed to high temperature equally and remove organic matter. The LOI determination was accomplished by following the methodology explained in [38], where empty crucibles were preheated at a temperature of 550°C for four hours, cooled in a desiccator, and weighed. Then, a mass of 200 mg of each sample was weighed in the cooled porcelain crucibles and left in the muffle furnace at a temperature of 550°C for four hours, removed from the furnace, cooled in desiccators for two hours, and weighed. The weight of the empty crucible and ignited samples were determined by using an ultrasensitive balance (Mettler Toledo, Model AB104-S). Weight differences were calculated to get LOI. The Certified Reference Standard (NCS DC 73027) from the China National Analysis Centre for Iron and Steel (Beijing) was used to validate the data.

### 2.4. Mineralogical Studies

To address the mineralogy of rock (thin section) samples, petrographic studies were conducted by visual estimation using a digital polarized microscope (ZEIS Model LR. 66238C and OPTIKA Model B-600POL-1) enhanced with image processing software Optica Vision. The ash mineralogy was determined using a binocular microscope (Model WILD M3C). These studies were carried out at the Geological Survey of Tanzania.

### 2.5. Particle Size and Shape

The particle size of the samples from the four sites (after drying at 105°C to constant weight) was determined according to the procedure described in [39]. The analysis was accomplished using a heavy-duty analytical sieve shaker controller (Model TMA-1170 with ANALYSETTE 18) loaded with different sieve sizes ranging from 0.045 mm to 2.00 mm. The sieve shaker controller was set to 10 minutes and a waiting interval of 5 seconds with an amplitude of 2.0. Shape classification was performed according to the procedure mentioned in [40], where samples were observed under the binocular microscope (Model WILD M3C). The shapes of the particles and counts were recorded.

### 2.6. Weather Data Collection

Temperature, pressure, humidity, rainfall, wind, direction, and speed, were monitored and recorded for one year (October 2020–September 2021) using a set of digital meteorological systems installed within the study site (2°59.614′S 35°21.068′E). The Youshiko digital weather station (Model YC9387 (W)) was used to collect the weather data. The digital weather station's specifications include barometric pressure measuring range of 525 to 825 mmHg at an operating temperature of −5 to 50°C, temperature readings in the range of −40 to 60°C with 0.6°C accuracy, wind speed range of 0 to 180 km/h with a resolution of 0.1 km/h, a 0 to 360°, sixteen wind direction measurement scale with a resolution of 1%, and relative humidity ranging from 20 to 99% with an accuracy of about 1% and rain gauge with an accuracy of 7%. The digital weather station was connected to the Internet via Wi-Fi, and data collection was monitored and downloaded from the host website https://www.wunderground.com.

## 3. Results

### 3.1. Elemental Composition

Chemical analysis of samples in SS was dominated by SiO_2_ (41.59–44.27 wt%), Al_2_O_3_ (5.97–10.78 wt%), FeO (total) (i.e., Fe^2+^ plus Fe^3+^) (11.61–13.12 wt%), CaO (15.17–16.39 wt%), MgO (8.14–10.16 wt%), and TiO_2_ (2.97–3.64 wt%). The analysis reveals the presence of trace elements like V, Mn, W, and Ba (Table 2). The chemical composition of ash taken from OTS west of OLA has the following concentrations: SiO_2_ (43.43–45.01 wt%), Al_2_O_3_ (9.14–10.74 wt%), FeO (total) (8.49–9.76 wt%), and CaO (12.79–13.75 wt%). Other oxides such as K and Ti have slightly lower values of 1.14–2.02 wt% and 2.24–2.99 wt%, respectively, with lower trace element values (Table 2). Volcanic ash taken one kilometer from the foot of OLA shows the concentrations of SiO_2_, FeO (total), Al_2_O_3_, and CaO are in the ranges of 41.22–42.75 wt%, 10.48–11.14 wt%, 6.83–7.25 wt%, and 14.41–15.15 wt%, respectively. Furthermore, TiO_2_ (2.81–3.45 wt%), K_2_O (0.89–1.14 wt%), and some trace elements are part of the chemical composition of ash (Table 2). Ash taken from the Ngorongoro crater shows high values of SiO_2_ (37.17–40.43 wt%), Al_2_O_3_ (9.62–11.62 wt%), FeO (total) (7.25–7.85 wt%), Na_2_O (11.94–12.51 wt%), and CaO (4.92–5.65 wt%). Other oxides such as Mg and Ti show slightly lower values of 8.12–9.54 wt% and 2.11–2.49 wt%, respectively, with lower values of trace elements, as indicated in Table 2. Furthermore, based on a cursory analysis, it appears that W, Ba, and Zr are good discriminants between OLA/OTS/SS and NCA.

### 3.2. Loss on Ignition

Samples from OTS, OLA, and NCA registered relatively higher values for LOI than those from SS. The order of LOI for different samples is NCA > OTS = OLA > SS (Table 2).

### 3.3. Mineralogy

**Figure 4:**
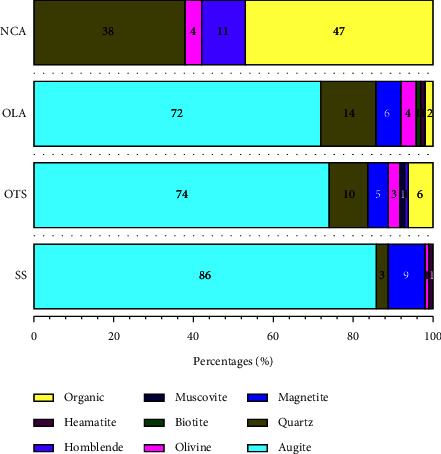
A plot of the average mineral proportions by the percentage of particles was determined by visual estimation in the samples taken from SS, OTS, OLA, and NCA.

The mineralogy of SS is dominated by augite (86% of particles), magnetite, quartz, and the remaining percentage by olivine and haematite. At the same time, the composition of OTS samples includes augite, magnetite, and quartz in a major percentage (89%) and olivine, muscovite, and biotite present in minor percentages. Samples from OLA showed the presence of augite, magnetite, and quartz in higher quantities (92%), and remaining olivine, biotite, muscovite, and haematite were present in lower quantities. NCA samples showed the presence of quartz, hornblende, and olivine. Generally, augite, quartz, and magnetite form more than 80% of the samples from OLA, OTS, and SS, while the rest of the sample proportions are olivine, biotite, and muscovite. The proportion of augite in the samples decreases from SS to OLA, while that of quartz decreases from OLA to SS. There is no augite in NCA samples, but there is a high proportion of quartz, hornblende, and olivine. NCA samples contain the highest percentage of organic matter. This result is also supported by the results obtained for LOI (Table 2). Muscovite and biotite are present in less than 1%, which cannot be seen in the graph due to a meagre percentage (Figure 4).

**Figure 5:**
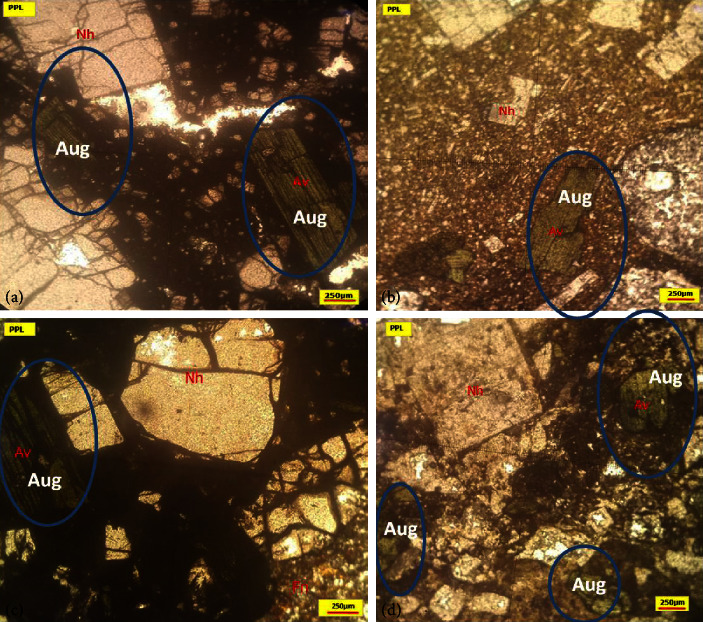
Petrographic microphotographs of the four rock samples taken from the foot of Oldoinyo Lengai (OLA).

Furthermore, a mineralogical examination of four rock samples collected from the foot of OLA reveals the presence of fine to coarse subhedral augite crystals that exist as matrices as well as phenocrysts (Figure 5). This study shows pyroxene signatures (augite, Av, or Aug) and nepheline (Nh).

### 3.4. Particle Size

**Figure 6:**
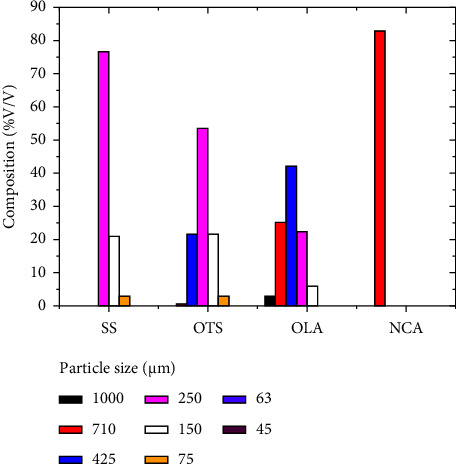
The particle size distribution of SS, OTS, OLA, and NCA samples.

**Figure 7:**
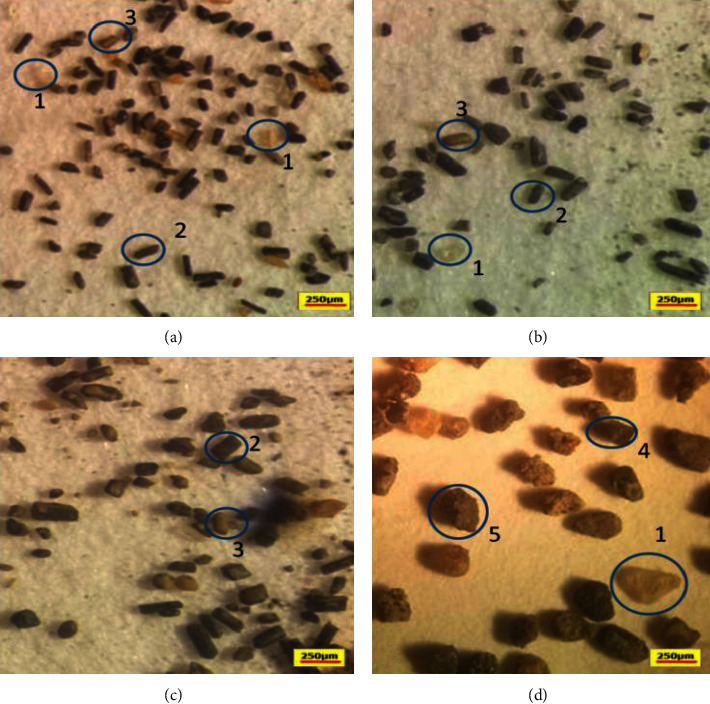
Images of the samples from the four sites (*A* = SS; *B* = OTS; *C* = OLA; *D* = NCA) under binocular microscopes that show ash size and major mineral types (1 = quartz; 2 = augite; 3 = magnetite; 4 = hornblende; 5 = heavily altered hornblende).

The particle size analysis results indicate that a large portion of SS comprises 250 *µ*m particles (76.91% by volume), followed by a particle size of 150 *µ*m (20.40%). The rest of the material constitutes small portions of other particle sizes (2.52%). The particle size of OTS showed that the materials are dominated by particles of 250 *µ*m (53.70%), followed by 450 *µ*m and 150 *µ*m (about 21.40% each). Only 2.38% of the material from OTS has a diameter of 75 *µ*m. The rest, about 0.63%, has a diameter of 710 *µ*m. On the other hand, an analysis of OLA shows significant variations in particle sizes ranging from 150 *µ*m to 1 mm, with the majority of the material at 425 *µ*m (42.50%), as shown in Figure 6. The particle size of NCA samples was dominated by 710 *µ*m (83.20%). The rest were organic debris which could not be accounted for this classification.  Figure 7 shows images of the ash material under the binocular microscope.

### 3.5. Particle Shape

**Figure 8:**
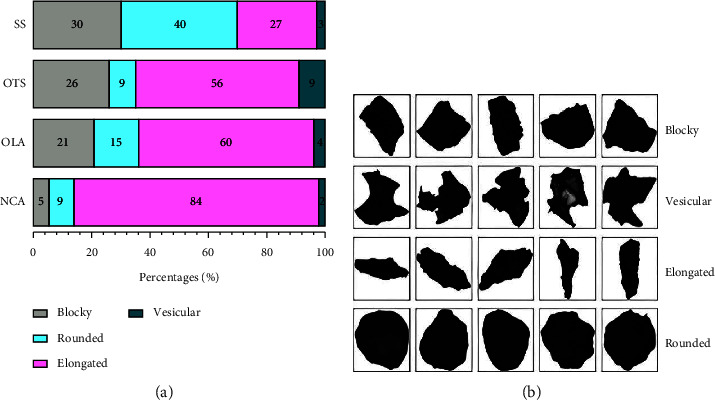
(a) The particle shape classification of the SS, OTS, OLA, and NCA samples. (b) The particle shape morphology shows blocky, vesicular, elongated, and rounded shapes [40].

Particle shape analysis of SS shows that most particles have an elongated shape. At the same time, there are an almost equal percentage of blocky and rounded-shaped particles with very few vesicular-shaped particles. In the samples of OTS, OLA, and NCA, sand is dominated by rounded-shaped particles, whereas in the case of SS, it is dominated by elongated particles. Particle shapes are represented in Figure 8.

### 3.6. Weather

**Figure 9:**
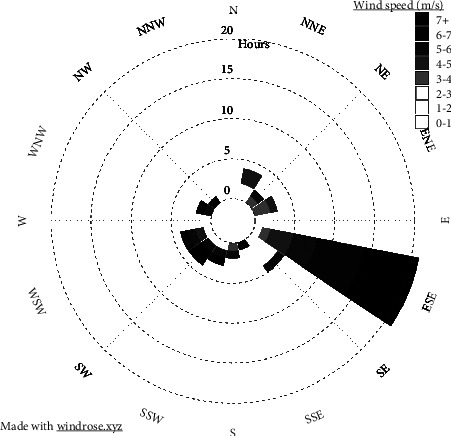
The wind rose presentation of the wind speed data in SS near the Oldupai Gorge area. The data were taken by using the Youshiko digital weather station from October 2020 to September 2021.

The monthly data (from October 2020 to September 2021) of weather elements gathered by using the Youshiko digital weather station on the study site registered the west-north-west (Figure 9) wind direction traveling at an average speed of 9.6 km/h. The maximum wind speed value attained during this period was 65.5 km/h, while the minimum speed was 0 km/h. An average temperature of 21.5°C and 64.4% humidity were registered. Annual precipitation was 669.5 mm.

## 4. Discussion

### 4.1. Elemental Composition

**Figure 10:**
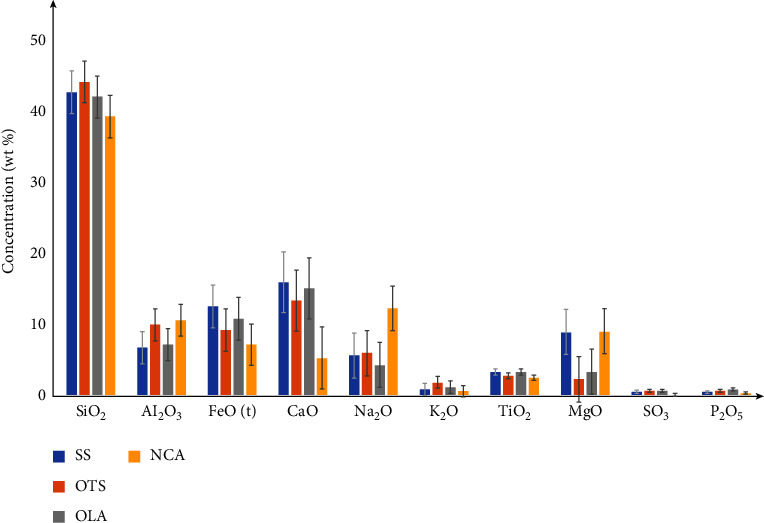
A graph of the average concentration of major oxides of SS, OTS, OLA, and NCA samples.

As described by previous researchers in tephrochronology and volcanology, various correlation tools have been applied in this study. These include the TAS diagram, CaO versus K_2_O plot, major oxide multiplots, and magnesian-ferriferous associations [22, 33]. Data in Table 2, which shows the elemental composition of samples from the four sites, are visualized in a grouped bar chart in Figure 10. Except for CaO, Na_2_O, and MgO, which vary slightly, all oxides from the four sample locations are substantially comparable.

**Figure 11:**
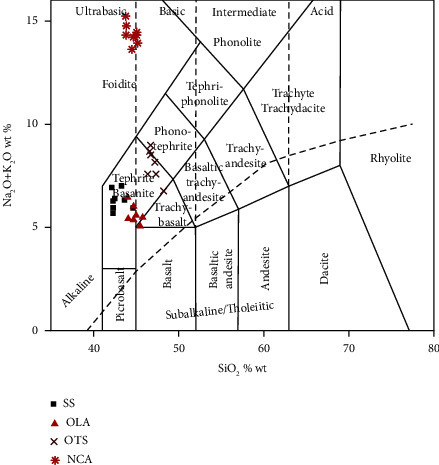
TAS classification diagram [33] of SS, OTS, OLA, and NCA samples plotted using GCDkit software [34].

TAS classification gives clues on the similarities of samples of volcanic origin taken from different locations in the same geographical area. It is an appropriate correlation tool in conjunction with other tools for searching for materials that originate from a common volcano [33]. When the elemental compositions from this study are plotted in a TAS classification diagram (Figure 11), SS, OTS, and OLA fall under the tephrite-basanite group. On the other hand, samples from NCA fall under the foidite group.

TAS classification generally indicates that there is a correlation between SS, OTS, and OLA samples. This suggests that the material (ash) may have originated from OLA's volcanic eruption. Ngorongoro lavas were described in earlier studies by Mollel [37]. Classification of lavas using a TAS plot placed lavas in basalt-trachyandesite, trachydacite, and trachydacite groups, contrary to the NCA samples. These variations may be due to following reasons: (1) the ash material (NCA) might have undergone mineralogical alterations due to environmental changes and (2) the Ngorongoro volcanic center may have produced numerous types of lava over time.

**Figure 12:**
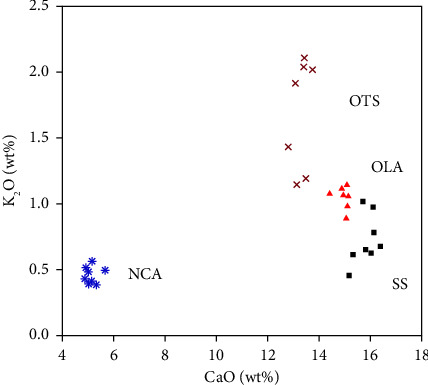
A plot of CaO against K_2_O of SS, OTS, OLA, and NCA samples.

A plot of CaO against K_2_O (Figure 12) for the identification of source volcanoes, as described in [23], shows a positive correlation between SS, OTS, and OLA, suggesting the materials are from a common source that could probably be from OLA. Differences in the concentrations of K_2_O in SS, OTS, and OLA samples can be due to the absence of easily weathered K-rich minerals such as biotite and muscovite, which have been lost as SS migrates along its path over the years [35]. Previous researchers reported changes in the chemical and mineral compositions of aeolian materials such as sand dunes, though the mechanisms of these changes are not fully understood [41]. The isolation of NCA samples on the left side of the plot is due to their low CaO content compared to SS, OTS, and OLA samples (Figure 12). The difference in CaO content shown on this plot is significant because it demonstrates agreement with previous studies on the nature of volcanic materials released from the OLA and Ngorongoro volcanic centers. Previous studies reported carbonatite material from OLA, which contains elevated amounts of up to 25 wt% CaO [9]. Geochemical studies of lava from the Ngorongoro volcanic center report low values of CaO, which range from 2 to 11 wt% [37]. Figure 12 shows that the SS, OLA, and OTS samples plot close to each other and far from the NCA samples, potentially reflecting a correlation between SS, OTS, and OLA. From this compositional comparison, SS materials most likely to have originated from OLA and NCA originated from the Ngorongoro volcanic center.

**Figure 13:**
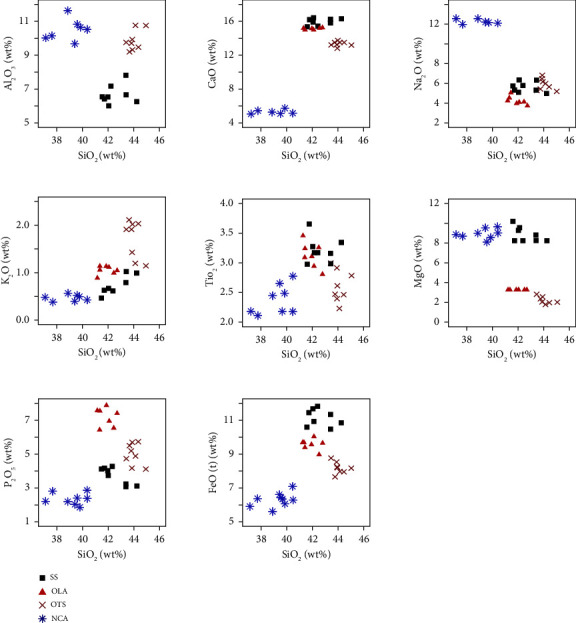
A multiplot of SiO_2_ against major oxides of SS, OTS, OLA, and NCA samples.

A multiplot of SiO_2_ against major oxides (Figure 13) shows correlations between SS, OTS, and OLA samples. The close agreement among these samples may be due to similarities in their mineralogical compositions, particularly in augite and magnetite (Figure 4). The isolation of the NCA samples suggests that they may have a different source from the other three (Figure 13). SS has been rolling for thousands of years [42]. As a result, the major glass component of the ash deposit (non-Fe/Mg-bearing silicate minerals such as quartz, feldspar, and muscovite) was easily weathered, leading to a gradual enrichment of SS in mafic minerals and increasing the concentration of FeO and MgO.

**Figure 14:**
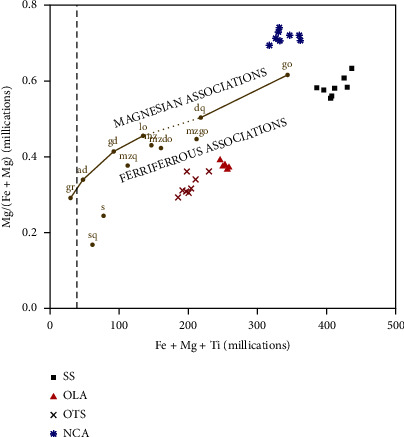
A plot of Fe + Mg + Ti against Mg/(Fe + Mg) as described in [43]. A line across the reference points, granite, adamellite, granodiorite, tonalite, quartz diorite, and gabbro is used to differentiate between magnesian and ferriferous associations. The dashed line presents leucogranites, which are granitic, light-colored igneous rocks with hardly any mafic minerals. Abbreviations: sq: quartz syenite, s: syenite, mzq: quartz monzonite, mz: monzonite, mzdq: quartz monzodiorite, mzgo: monzogabbro.

Iron, titanium, and magnesium elements are used to distinguish ferriferous and magnesian magmatic series samples by plotting Mg/(Fe + Mg) against Fe + Mg + Ti [43]. The distribution patterns between magnesian and ferriferous magmatic series for samples from SS, OTS, OLA, and NCA are shown in Figure 14. The separation on a plot is based on the position of the reference line passing through granite, adamellite, granodiorite, tonalite, quartz diorite, and gabbro points. Except for the samples from the Ngorongoro crater (NCA), the plot revealed that all the samples (SS, OTS, and OLA) in this study belong to the ferriferous magmatic series [33]. Magnesiohornblende in the NCA samples (Figure 4) partly contributed to magnesian magmatic nature. The presence of the magnesium-rich foidite lava group and Mg-rich olivine has been reported in the Ngorongoro crater [37, 44]. The distribution pattern of the samples suggests there are the existing similarities between SS, OTS, and OLA. Generally, results of the elemental composition of ash material in this study show a strong indication that OLA is the most likely source of both SS and OTS.

### 4.2. Loss on Ignition

LOI is attributed to organic contaminants from vegetation mixed with ash materials and accumulating over time. Samples from SS have very low values of LOI compared to other samples. SS is constantly in motion, being blown westward. As a result, the accumulation of any organic contaminant is restricted due to density differences between minerals in ash and organic matter, hence, very low values of LOI (Table 2). On the other hand, samples from NCA have the highest values of LOI. The highest values of LOI for the samples from NCA relate to the accumulation of organic matter over time, which defines materials' age.

### 4.3. Mineralogy

The polarising microscope reveals blocky and elongated-shaped augite, which is similar to that found in ash samples from SS, OTS, and OLA. Examination of thin section slides showed the prevalence of nepheline minerals which do not appear in all ash samples for unknown reasons that need further studies or could not be erupted at that time [9, 45] but could be due to fractionation by density effects during transport and by variations of magmatic compositions over time, which are common in volcanic eruption history of many volcanic centers [9, 46].

Mineralogical studies of SS show the domination of augite (86%), magnetite, and quartz minerals (Figure 4). Augite is a member of the pyroxene group of rock-forming minerals and is normally associated with an elongated crystalline shape at the particle level. The presence of augite with the chemical formula (Ca_x_,Mg_y_,Fe_z_) (Mg_y1_,Fe_z1_) Si_2_O_6_ (where 0.4 ≤ *x* ≤ 0.9, *x* + *y* + *z* = 1 and *y*_1_+*z*_1_ = 1) [47] is reflected in the chemical composition of SS by the presence of elevated values of oxides of Si, Ca, Fe, and Mg. According to a study by Sigamony [48] on magnetic properties, augite has anisotropic properties at low fields and isotropic properties at high fields. These observations are in line with the ferromagnetic behavior observed in the SS, OTS, and OLA samples, as their particles are strongly attracted to the magnetic field. Titanium oxide (2.97 to 3.64%) in SS (Table 2) can be attributed to the presence of titaniferous magnetite minerals with the chemical formula Fe^2+^(Fe^3+^, Ti)_2_O_4_). Other elements such as zinc, manganese, chromium, nickel, and vanadium appear as common inclusions in magnetite and augite minerals.

Augite, quartz, magnetite, olivine, biotite, muscovite, and haematite minerals have also been observed in OTS and OLA (Figure 4). Observations of augite, quartz, and magnetite reveal mineralogical similarities between SS, OTS, and OLA. The three sites are geographically aligned from east to west (Figure 1), hypothesizing the spread of these materials in the east-west direction. A significant difference shown by NCA mineralogy is the absence of augite and the presence of hornblende minerals. Previous studies on the mineralogy of volcanic rocks (lava) at NCA indicate the presence of minor augite and biotite minerals [37], which may have been altered and not detected in ash in this study. Therefore, the distinguishable mineralogical composition of ash material from NCA supports the interpretation of the chemical and rock petrology data that SS around Oldupai Gorge may have originated not from activities during or after the formation of the Ngorongoro crater but rather from the volcanic activities of OLA.

### 4.4. Particle Size

As shown in Figures 6 and 7, the proportion of 250 *µ*m particles increases from east to west (i.e., from OLA to SS). Fine-grained and lighter particles were ejected and transported by the wind to a considerable distance [21]. This pattern is consistent with the tendency for coarse-grained ash material to be deposited closer to the source volcano due to more rapid gravitational settling of larger particles.

### 4.5. Particle Shape

Particle shape examination of SS, OTS, and OLA show the presence of blocky, elongated, rounded, and vesicular shapes (Figures 7 and 8). Blocky and elongated shapes are mostly associated with augite (Figure 5), which have been observed in SS, OTS, and OLA (Figure 4). The proportion of blocky and vesicular-shaped ash in OTS is higher than that in OLA. This is because the sampling at OLA occurred in streamsides and valleys where the material is mostly exposed. There is a high probability that the blocky and vesicular ash material could have been washed out with time (because of shapes). Conversely, vegetative dunes in the OTS area (Figure 2(b)) likely contribute to the preservation of ash. Comparing the morphology of ash allows one to infer whether samples originated from a common volcano. Since blocky and elongated ash dominated in augite, OLA was the most probable source. Other shapes (like rounded) were a mixture of varieties of minerals.

Chemical and mineralogical studies of the ash material in this study suggest that they result from volcanic eruptions from the Gregory Rift volcanoes. The Gregory Rift volcanoes include a series of volcanic cones around Ngorongoro along the east arm of the rift valley. The mineral content of the ash material released by volcanoes from the area [49–52] is similar to the ash material in this study. Perhaps the most important question is which peak was the source volcano of the materials under investigation. To answer this question, a chronological account of the region's volcanism needs to be addressed. All volcanic peaks in the Gregory Rift ceased their activities around 2 Ma ago except for OLA, which continues its activities to date [53]. The region's chronology indicates that SS formed following the volcanic eruption of one of the Gregory Rift volcanoes. Chronology data of the area, the nature of the SS material, and its dune mobility behavior rule out the possibility of these materials originating from volcanic peaks of the Gregory Rift other than currently active OLA.

### 4.6. Weather

**Figure 15:**
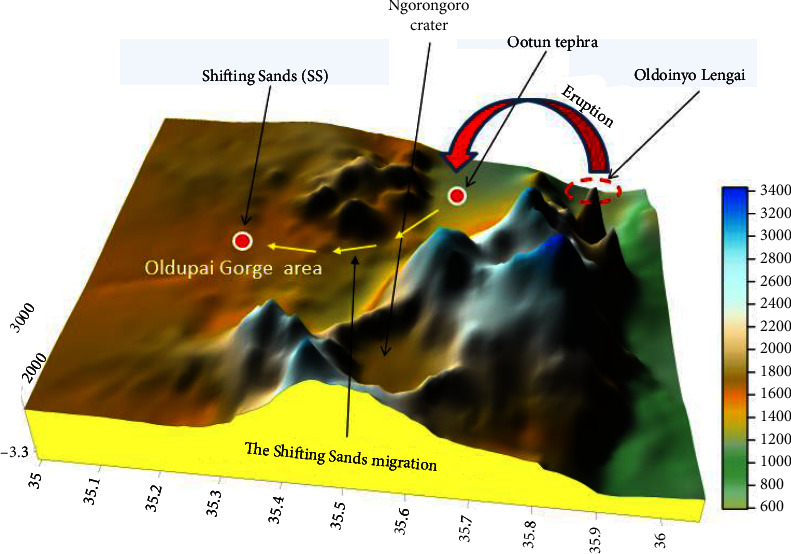
A model of the study site was generated from the Shuttle Radar Topography Mission (SRTM) digital elevation dataset collected in 2000, using Surfer software version 16.

The general west wind direction is consistent with the current (2020) direction of SS and aligns with the geographical features from OLA (Figure 15). The weather data collected over one year (at the Oldupai Gorge area) indicated that the wind blows in a west-north-west direction (Figure 9). The wind travels at an average speed of 9.6 km/h and provides sufficient force to blow the particles of SS in the east-west direction across the flat corridor from the Ootun area to the Oldupai Gorge area (Figure 15). The model shows SS migration from the OTS area to the Oldupai Gorge area, which takes thousands of years [42]. SS has been derived from Ootun tephra deposition, which resulted from OLA eruption. The OTS location is roughly 35 km west of OLA and about 45 km east of SS. The historical east-west wind direction [9, 51] supports the hypothesis that volcanic ash that erupted at OLA traveled by the action of wind and was deposited several kilometers away in the west. Lighter and smaller particles were deposited far west, while heavier and coarser particles were deposited closer to the OLA source volcano. The observations from weather data in this study agree with the results obtained for particle size and ash distribution patterns across the study site.

## 5. Conclusions

Based on the correlation of both geochemical and geomorphological properties of the material, SS ash, OTS ash, and OLA ash seem to contain more augite than any mineral. The study suggests that the ash materials that constitute SS and OTS have attributes of tephra material from past volcanic eruptions of the same origin. Oldoinyo Lengai, an active volcano east of Oldupai Gorge, has been implicated as a possible source of this tephra material. Significant amounts of tephra depositions observed in OTS located west of OLA are evidence of the magnitude of volcanic plumes, leading to the formation of present tephra bedforms in the region. Depositions are suggested to have been laid down across the western part of OLA and set in motion as sand dunes for possibly hundreds to thousands of years, of which many were dispersed on the plain and no longer exist. Therefore, SS materials are suggested to have been brought from the Ootun area to the Oldupai Gorge region by the action of wind in the direction of west-south-west through the ground channel. The tephra material at Ootun was heavy enough to be carried away by the wind and form barchan dunes. Eruptive periods of OLA, however, remain a subject of further speculation, which may involve proximal tephrostratigraphy of OTS with an additional chronology component and ultimately unravel time-based tephra dispersal patterns across the region. Data enrichment from these topics could further fine-tune conclusions deduced from this study.

## Figures and Tables

**Table 1:** Description of samples collected in the study.

S/no.	Sample name	No. of samples	Geographical location	Description	Analysis methods
1	Shifting sands (SSs)	8	Shifting sands (2°56′32″S 35°18′42″E)	Particles	XRF, muffle furnace, sieve shaker, binocular, and petrographic microscopy analysis
2	Ootun sands (OTSs)	8	Ootun sands (2°46′44″S 35°36′16″E)	Particles	
3	Oldoinyo Lengai (OLA)	8	Oldoinyo Lengai (2°45′34″S 35°54′50″E)	Particles	
4	Ngorongoro Conservation Area (NCA)	8	Ngorongoro crater (3°13′08″S 35°31′31″E)	Particles	
5	Igneous rocks (ijolite) from OLA	4	Oldoinyo Lengai (2°45′34″S 35°54′50″E)	Rocks	Petrographic microscopy
Total samples	**36**				

**Table 2:** Major and minor oxides (wt%) and trace elements (ppm) of SS, OTS, OLA, and NCA samples (EDXRF data are not normalized).

	SiO_2_	Al_2_O_3_	FeO (*t*)	CaO	Na_2_O	K_2_O	TiO_2_	MgO	SO_3_	P_2_O_5_	LOI	V	Mn	Ni	Zn	As	W	Cu	Ba	Y	Zr
	wt%											Ppm									
SS 01	42.12	5.97	12.12	16.39	6.22	0.67	3.17	9.45	0.34	0.38	0.89	812	2071	39	162	58	1.78	55	626	37	537
SS 02	43.43	6.57	12.57	15.71	5.24	1.01	2.97	8.78	0.39	0.30	1.05	804	2079	34	167	48	1.79	49	630	35	530
SS 03	41.59	6.48	11.77	15.17	5.68	0.45	2.97	10.16	0.41	0.41	1.09	824	2055	41	155	55	1.64	46	647	39	525
SS 04	43.44	7.76	11.61	16.16	6.22	0.78	3.14	8.14	0.31	0.32	1.11	829	2049	34	152	65	1.55	54	655	47	528
SS 05	44.27	6.16	12.01	16.13	4.89	0.97	3.33	8.21	0.33	0.31	1.05	824	2068	41	189	57	1.45	57	658	48	549
SS 06	42.04	6.49	13.01	15.81	4.98	0.65	3.26	9.17	0.48	0.40	0.85	820	2020	42	164	48	1.89	61	624	41	573
SS 07	42.41	7.11	13.12	15.33	5.73	0.61	3.17	8.16	0.49	0.42	0.94	822	2089	48	184	47	1.84	65	635	43	544
SS 08	41.76	6.37	12.73	16.03	5.24	0.62	3.64	8.14	0.37	0.41	0.97	810	2054	39	158	49	1.44	57	641	47	541
OTS 01	44.12	10.72	8.87	13.48	5.88	1.19	2.24	1.84	0.24	0.49	5.86	149	2351	116	205	49	1.08	105	1086	34	609
OTS 02	43.91	9.89	9.11	13.75	6.41	2.02	2.62	2.05	0.41	0.42	5.75	121	2298	124	195	45	1.22	120	968	45	564
OTS 03	43.88	9.69	9.17	13.07	6.11	1.91	2.92	2.14	0.46	0.52	4.85	145	2248	136	145	48	1.45	142	956	45	582
OTS 04	43.71	9.14	8.49	13.42	5.45	2.11	2.47	2.41	0.56	0.55	5.42	162	2298	156	168	67	1.65	163	987	41	612
OTS 05	44.41	9.45	8.83	13.41	5.63	2.04	2.47	2.01	0.47	0.58	5.68	145	2314	122	157	59	1.35	125	1021	46	632
OTS 06	45.01	10.74	9.13	13.14	5.14	1.14	2.79	2.02	0.56	0.41	5.63	124	2365	141	175	58	1.24	111	1045	42	624
OTS 07	43.43	9.73	9.76	13.11	5.19	1.91	2.99	2.78	0.78	0.48	4.44	155	2395	178	164	47	1.45	123	1033	47	598
OTS 08	43.89	9.27	9.48	12.79	6.75	1.43	2.38	2.47	0.72	0.57	4.89	133	2244	154	146	64	1.32	124	1092	48	574
OLA 01	42.11	7.11	11.14	14.88	4.12	1.11	2.94	3.23	0.49	0.70	4.45	126	2911	28	289	46	1.19	105	1700	59	679
OLA 02	41.91	6.83	10.63	15.09	3.93	1.14	3.11	3.23	0.58	0.79	4.65	153	2916	22	279	41	1.14	95	1717	47	817
OLA 03	42.11	6.96	10.62	14.41	3.82	1.07	3.21	3.23	0.41	0.66	5.05	147	2844	24	289	31	1.19	87	1817	68	645
OLA 04	41.43	7.25	10.48	14.95	5.01	1.06	3.24	3.23	0.43	0.75	3.89	185	2719	19	297	39	1.28	112	1845	59	712
OLA 05	42.75	7.11	10.72	15.15	3.71	1.05	2.81	3.23	0.39	0.74	4.11	165	2990	35	212	29	1.27	91	1847	49	634
OLA 06	41.38	7.02	10.76	15.08	4.47	1.14	3.09	3.23	0.44	0.64	5.56	147	2849	31	254	49	1.24	102	1791	47	711
OLA 07	41.22	7.06	10.78	15.07	4.16	0.89	3.45	3.23	0.49	0.76	5.78	195	2844	29	271	55	1.23	117	1844	52	694
OLA 08	42.49	7.12	9.96	15.10	4.12	0.98	3.26	3.23	0.54	0.66	5.44	158	2838	22	249	35	1.11	87	1746	55	621
NCA 01	38.87	11.62	6.25	5.16	12.51	0.56	2.45	8.98	0.00	0.22	11.55	619	1415	110	121	59	0.81	110	685	65	448
NCA 02	39.81	10.66	6.78	5.65	12.14	0.49	2.49	8.49	0.00	0.19	11.48	652	1411	98	148	46	0.86	98	655	45	389
NCA 03	37.17	9.98	6.55	5.02	12.47	0.48	2.19	8.89	0.00	0.22	13.15	635	1369	59	146	48	0.45	66	698	14	358
NCA 04	39.41	9.64	7.32	5.03	12.12	0.39	2.65	9.51	0.00	0.20	11.47	695	1357	69	495	47	0.75	95	589	45	346
NCA 05	37.71	10.11	7.11	5.32	11.94	0.38	2.11	8.69	0.00	0.26	12.86	685	1324	101	124	49	0.96	78	548	49	318
NCA 06	40.41	10.46	7.85	5.14	11.96	0.41	2.17	9.54	0.00	0.29	11.32	639	1396	98	129	43	1.12	85	576	46	396
NCA 07	40.43	10.54	6.99	4.87	12.04	0.43	2.77	9.05	0.00	0.24	12.09	647	1414	119	136	39	0.86	74	618	39	415
NCA 08	39.59	10.78	7.07	4.92	12.15	0.51	2.19	8.12	0.00	0.24	12.52	651	1425	99	128	32	0.92	95	634	55	399

## Data Availability

All the data used to support the findings of this study are included in the article.

## References

[1] Livingstone I. (1990). Desert sand dune dynamics: review and prospect. *Namib Ecology: 25 Years of Namib Research, Transvaal Museum Monograph*.

[2] Tsoar H. (2001). Types of aeolian sand dunes and their formation. *Geomorphological Fluid Mechanics, Lecture Notes in Physics*.

[3] Reffet E., Courrech du Pont S., Hersen P., Douady S. (2010). Formation and stability of transverse and longitudinal sand dunes. *Geology*.

[4] Chojnacki M., Moersch J. E., Burr D. M. (2010). Climbing and falling dunes in valles marineris, mars. *Geophysical Research Letters*.

[5] Edgett K. S., Lancaster N. (1993). Volcaniclastic aeolian dunes: terrestrial examples and application to martian sands. *Journal of Arid Environments*.

[6] Fiske R. S., Rose T. R., Swanson D. A., Champion D. E., McGeehin J. P. (2009). Kulanaokuaiki Tephra (ca. AD 400–1000): newly recognized evidence for highly explosive eruptions at Kilauea Volcano Hawai’I. *The Geological Society of America Bulletin*.

[7] Neukirchen F., Finkenbein T., Keller J. (2010). The lava sequence of the east african rift escarpment in the Oldoinyo Lengai–lake natron sector, Tanzania. *Journal of African Earth Sciences*.

[8] Wiedenmann D., Keller J., Zaitsev A. N. (2010). Melilite-group minerals at oldoinyo lengai, Tanzania. *Lithos*.

[9] Sherrod D. R., Magigita M. M., Kwelwa S. (2013). *Geologic Map of Oldoinyo Lengai (Oldoinyo Lengai) Volcano and Surroundings, Arusha Region*.

[10] Mana S., Furman T., Carr M. (2012). Geochronology and geochemistry of the essimingor volcano: melting of metasomatized lithospheric mantle beneath the north Tanzanian divergence zone (east african rift). *Lithos*.

[11] Fairhead J. D. (1980). The structure of the cross-cutting volcanic chain of Northern Tanzania and its relation to the East African rift system. *Tectonophysics*.

[12] Dawson J. B. (1992). Neogene tectonics and volcanicity in the North Tanzania sector of the gregory rift valley: contrasts with the Kenya sector. *Tectonophysics*.

[13] Keller C. M., Hansen C., Alexander C. S. (1975). Archaeology and paleoenvironments in the manyara and engaruka basins, Northern Tanzania. *Geographical Review*.

[14] Ryan W. B. F., Carbotte S. M., Coplan J. O. (2009). Global multi-resolution topography synthesis. Geochem. *Geophysic Geosystem*.

[15] Hay R. L. (1976). *Geology of the Olduvai Gorge*.

[16] Lowe D. J. (2011). Tephrochronology and its application: a review. *Quaternary Geochronology*.

[17] Geyer A., Marti A., Giralt S., Folch A. (2017). Potential ash impact from Antarctic volcanoes: insights from Deception Island’s most recent eruption. *Scientific Reports*.

[18] Langmann B. (2013). Volcanic ash versus mineral dust: atmospheric processing and environmental and climate impacts. *International Scholarly Research Notices*.

[19] Pyle D. M. (2015). *Field Observations of Tephra Fallout Deposits*.

[20] Plunkett G., Coulter S. E., Ponomareva V. V., Blaauw M., Klimaschewski A., Hammarlund D. (2015). Distal tephrochronology in volcanic regions: challenges and insights from Kamchatkan lake sediments. *Global and Planetary Change*.

[21] Durant A. J., Villarosa G., Rose W. I., Delmelle P., Prata A. J., Viramonte J. G. (2012). Long-range volcanic ash transport and fallout during the 2008 eruption of Chaitén volcano, Chile. *Physics and Chemistry of the Earth, Parts A/B/C*.

[22] Shane P. (2000). Tephrochronology: a New Zealand case study. *Earth-Science Reviews*.

[23] Lowe D. J., Shane P. A. R., Alloway B. V., Newnham R. M. (2008). Fingerprints and age models for widespread New Zealand tephra marker beds erupted since 30, 000 years ago: a framework for NZ-INTIMATE. *Quaternary Science Reviews*.

[24] Dugmore A. J., Larsen G., Newton A. J. (2004). *Tephrochronology and its Application to Late Quaternary Environmental Reconstruction, with Special Reference to the North Atlantic Islands*.

[25] Turney C. S. M., Lowe J. J., Davies S. M. (2004). Tephrochronology of last termination sequences in Europe: a protocol for improved analytical precision and robust correlation procedures (a joint SCOTAV-INTIMATE proposal). *Journal of Quaternary Science*.

[26] Barrell D. J. A., Alloway B. V., Shulmeister J. (2005). Towards a climate event stratigraphy for New Zealand over the past 30, 000 years. *Scientific Reports*.

[27] Katsui Y., Suzuki T., Soya T. (1989). *Geological Map of Hokkaido-Komagatake Volcano. Geological Map of Volcano 5*.

[28] Alloway B. V., Larsen G., Lowe D. J. (2006). *Tephrochronology*.

[29] Gale S. J. (2009). Dating the recent past. *Quaternary Geochronology*.

[30] Maters E. C., Delmelle P., Rossi M. J., Ayris P. M., Bernard A. (2016). Controls on the surface chemical reactivity of volcanic ash investigated with probe gases. *Earth and Planetary Science Letters*.

[31] Delmelle P., Wadsworth F. B., Maters E. C., Ayris P. M. (2018). High temperature reactions between gases and ash particles in volcanic eruption plumes. *Reviews in Mineralogy and Geochemistry*.

[32] Nakagawa M., Ohba T. (2003). Minerals in volcanic ash. 1: primary minerals and glass. *Global Environmental Research*.

[33] Bas M. J. L., Maitre R. W. L., Streckeisen A., Zanettin B. (1986). A chemical classification of volcanic rocks based on the total alkali-silica diagram. *Journal of Petrology*.

[34] Janoušek V., Farrow C. M., Erban V. (2006). Interpretation of whole-rock geochemical data in igneous geochemistry: introducing geochemical data toolkit (GCDkit). *Journal of Petrology*.

[35] Kafumu D. P. (2020). The geochemical compositions and origin of sand dunes in the Olduvai Gorge–Eastern Serengeti plains, Northern Tanzania. *Tanzania Journal of Science*.

[36] Bell K., Simonetti A. (1996). Carbonatite magmatism and plume activity: implications from the Nd, Pb and Sr isotope systematics of Oldoinyo Lengai. *Journal of Petrology*.

[37] Mollel G. F., Swisher C. C., Feigenson M. D., Carr M. J. (2008). Geochemical evolution of Ngorongoro caldera, northern Tanzania: implications for crust–magma interaction. *Earth and Planetary Science Letters*.

[38] Wright A. L., Wang Y., Reddy K. R. (2008). Loss-on-ignition method to assess soil organic carbon in calcareous everglades wetlands. *Communications in Soil Science and Plant Analysis*.

[39] Nogami K., Hirabayashi J. I., Nishimura Y., Suzuki A. (2002). Nature and origin of volcanic ash in the 2000 eruption of usu volcano, Southwestern Hokkaido, Japan. *Earth Planets and Space*.

[40] Shoji D., Noguchi R., Otsuki S., Hino H. (2018). Classification of volcanic ash particles using a convolutional neural network and probability. *Scientific Reports*.

[41] Craddock R. A. (2011). Aeolian processes on the terrestrial planets: recent observations and future focus. *Progress in Physical Geography: Earth and Environment*.

[42] Belsky A. J., Amundson R. G. (1986). Sixty years of successional history behind a moving sand dune near Olduvai Gorge, Tanzania. *Biotropica*.

[43] Debon F., Le Fort P. (1988). A cationic classification of common plutonic rocks and their magmatic associations. Principles, methods, applications. *Bulletin de Mineralogie*.

[44] Mollel G. F. (2002). *Petrochemistry and Geochronology of Ngorongoro Volcanic Highland Complex (NVHC) and its Relationship to Laetoli and Olduvai Gorge, Tanzania”, Masters Thesis*.

[45] Dawson J. B. (1998). Peralkaline nephelinite-natrocarbonatite relationships at Oldoinyo Lengai, Tanzania. *Journal of Petrology*.

[46] Gertisser R., Keller J. (2003). Temporal variations in magma composition at merapi volcano (Central Java, Indonesia): magmatic cycles during the past 2000 years of explosive activity. *Journal of Volcanology and Geothermal Research*.

[47] Morimoto N., Fabries J., Ferguson A. K. (1988). Nomenclature of pyroxenes. *American Mineralogist*.

[48] Sigamony A. (1945). Magnetic properties of augite. *Springer*.

[49] Church A. A., Jones A. P. (1995). Silicate—carbonate immiscibility at oldoinyo lengai. *Journal of Petrology*.

[50] Keller J., Krafft M. (1990). Effusive natrocarbonatite activity of oldoinyo lengai, june 1988. *Bulletin of Volcanology*.

[51] Hay R. L. (1983). Natrocarbonatite tephra of Kerimasi volcano, Tanzania. *Geology*.

[52] Mariano A. N., Roeder P. L. (1983). Kerimasi: a neglected carbonatite volcano. *The Journal of Geology*.

[53] Żaba J., Gaidzik K. (2011). The Ngorongoro crater as the biggest geotouristic attraction of the gregory rift (Northern Tanzania, Africa)–geological heritage. *Geotourism/Geoturystyka*.

